# Use of Contrast-Enhanced Ultrasonography for the Characterization of Tumor Thrombi in Seven Dogs

**DOI:** 10.3390/ani10091613

**Published:** 2020-09-10

**Authors:** Alessia Cordella, Pascaline Pey, Nikolina Linta, Manuela Quinci, Marco Baron Toaldo, Luciano Pisoni, Giuliano Bettini, Alessia Diana

**Affiliations:** 1 Department of Veterinary Medical Sciences, School of Veterinary Medicine, University of Bologna, 40126 Bologna, Italy; alessia.cordella@outlook.com (A.C.); nikolina.linta2@unibo.it (N.L.); manuela.quinci2@unibo.it (M.Q.); luciano.pisoni@unibo.it (L.P.); giuliano.bettini@unibo.it (G.B.); alessia.diana@unibo.it (A.D.); 2 Department of Medical Imaging of Domestic Animals, Faculty of Veterinary Medicine, Ghent University, 9820 Merelbeke, Belgium; 3 Division of Cardiology, Clinic for Small Animal Internal Medicine, Vetsuisse Faculty University of Zürich, CH-8057 Zürich, Switzerland; marco.barontoaldo@uzh.ch

**Keywords:** canine, contrast medium, SonoVue, oncology, ultrasound

## Abstract

**Simple Summary:**

Some neoplasia—such as adrenal and thyroid glands tumors—have been associated with “tumor thrombi” both in humans and dogs. The detection and characterization of these venous tumor thrombi is important for both surgical planning and prognosis. In human medicine, contrast-enhanced ultrasonography (CEUS) is considered an accurate diagnostic technique for differentiating malignant from benign portal vein thrombosis in hepatocellular carcinomas. Data regarding the characteristics of tumor thrombi in dogs are currently lacking. Therefore, the aim of this study was to assess the feasibility of CEUS for the characterization of malignant venous thrombosis in dogs. On the basis of our results, CEUS appeared to be useful in the detection of malignant intravascular invasion; contrast uptake of the thrombus was present in all cases. In addition, CEUS may clearly detect newly formed vessels within the thrombus, and arterial-phase enhancement and washout in the venous phase were the main features in malignant thrombosis in our dogs. As CEUS is an easy to perform, noninvasive technique, its application in the detection of malignant thrombosis in dogs may be used to improve the diagnosis in oncological canine patients.

**Abstract:**

Tumors of adrenal and thyroid glands have been associated with vascular invasions—so-called tumor thrombi, both in humans and dogs. The detection and characterization of venous thrombi is an important diagnostic step in patients with primary tumors for both surgical planning and prognosis. The aim of this study was to describe the use of contrast-enhanced ultrasonography (CEUS) for the characterization of tumor thrombi. Dogs with tumor thrombus who underwent bi-dimensional ultrasound (B-mode US) and CEUS were included. Seven dogs were enrolled in this retrospective case series. On B-mode US, all thrombi were visualized, and vascular distension and thrombus-tumor continuity were seen in three and two cases, respectively. On color Doppler examination, all thrombi were identified, seemed non-occlusive and only two presented vascularity. On CEUS, arterial-phase enhancement and washout in the venous phase were observed in all cases. Non-enhancing areas were identified in the tumor thrombi most likely representing non-vascularized tissue that could potentially be embolized in the lungs after fragmentation of the tumor thrombi. On the basis of these preliminary study, CEUS appeared to be useful for the characterization of malignant intravascular invasion.

## 1. Introduction

Tumor thrombi are defined as tumors extending into a vessel—typically a vein. It is crucial to distinguish tumor thrombi from non-malignant thrombi in the setting of neoplasia, as this often impacts staging and treatment approaches [1,2,3,4,5,6]. In particular, thyroid carcinomas have the tendency to infiltrate adjacent structures (including vessels), and the risk of metastases increases when the thyroid vein is occupied by a neoplastic thrombus [4,6]. Tumors of the adrenal glands have been associated with tumor thrombi in humans and dogs [1,2,3,5]; previous studies reported that dogs with caval tumor thrombi may have a poorer long-term prognosis [3,7]. In addition, the invasion of the caudal vena cava is associated with higher postoperative mortality rate—especially when invasion is extensive (thrombi extending cranial to the hepatic hilus) [5]. Fast and less invasive surgical techniques have been recently described in dogs with caval invasion, with good results [8,9], despite the presence of a post-hepatic thrombus or long caval occlusion time [9]. According to another recent study, dogs with post-diaphragmatic tumor thrombi still showed a worse prognosis [10]. Therefore, detection and characterization of tumor thrombi is of great importance in patients with primary tumors for both surgical planning and prognosis.

Ultrasound examination is fast and less invasive method to examine intra-abdominal structure, thyroid lobes and adjacent vessels, showing high sensitivity (100%) and specificity (96%) in detecting vascular invasion [11]. In another study concerning adrenal tumors, the sensitivity and specificity of abdominal US for the detection of a caval thrombus were 80 and 90%, respectively [3], while contrast-enhanced CT showed 92% sensitivity and 100% specificity for intraluminal invasion from adrenal masses in dogs [12]. Color flow Doppler (CFD) examination of the intravascular formation can be useful in diagnosing tumor thrombi, but the vascularization is often so poor that no signal can be seen inside the lesion [13,14]. In humans, contrast-enhanced ultrasonography (CEUS) is considered an accurate diagnostic technique for the characterization of portal vein thrombosis complicating hepatocellular carcinoma, with high sensitivity (88–97.7%), 100% specificity and very good accuracy (92.5–95.5%) in distinguishing benign from tumor thrombi [13,14,15,16,17]. In dogs, the presence of thrombosis associated with adrenal and thyroid masses is most likely to be malignant, as these tumors have the tendency of invade the vessels and adjacent tissues [1,2,3,4,5,6,7]. Nevertheless, CEUS could be useful in these cases to confirm the anatomic continuity with the tumor and to assess the homogeneity of the tumor thrombus in order to anticipate the possibility of fragmentation, which could lead to pulmonary thromboembolism and/or could represent a surgical complication, as previously described [10]. Despite the high sensitivity and specificity of CT in detecting vascular invasion in dogs [11], this modality may not be performant in the characterization of the homogeneity of the thrombus and also for detection of potential endothelial invasion, because of frequent streamlining artefacts and lesser spatial resolution compared to CEUS. For these reasons, CEUS could represent a useful technique for the detection and characterization of tumor thrombi in dogs.

The purpose of this study was to describe CEUS features of tumor thrombi in dogs, with particular attention to the risk of fragmentation and endothelial invasion and compare them with their conventional ultrasound (US) and CFD characteristics.

## 2. Materials and Methods

Medical records of dogs admitted to the Veterinary Teaching Hospital of the University of Bologna from January 2012 through January 2016 were reviewed and dogs with primary masses and associated venous thrombi were selected. Dogs were included in the study if they had a complete US examination of the mass and associated thrombus, including B-mode US, CFD examination and CEUS. Only dogs with a cytological or histological diagnosis of the primary mass, associated venous thrombus or both were selected.

The following information were recorded for each case: breed, sex, age, anamnesis, results of physical examination, laboratory tests and histological or cytological findings.

Two-dimensional US, CFD and CEUS images were reviewed off-line. Bi-dimensional findings recorded were: Localization, shape, dimension, echostructure of the primary lesion, presence of intravascular thrombus showing anatomic continuity with the primary lesion and dilation of the invaded vessel. The evaluated CFD findings were: Presence of residual flow within the invaded vessel and presence of intralesional signal within the primary lesion and the thrombus. Finally, CEUS findings evaluated were: contrast uptake (present or absent), aspect of the contrast uptake (homogeneous/heterogeneous), margination of the thrombus (regular and well-defined/irregular and ill-defined), comparison between timing in contrast uptake by the thrombus and the invaded vessel and comparison between timing in contrast uptake by the thrombus and the primary lesion.

Histological or cytological findings of the primary tumor or of the thrombus, when available, were recorded. Samples were collected by ultrasound-guided fine needle aspiration (FNA) and/or tissue core biopsy (TB) or at necropsy.

## 3. Results

Seven dogs met the inclusion criteria. The median age was 11 (7–13) years, and the median body weight was 27 (9–33) kg. Information regarding the signalment and clinical signs are summarized in Table 1.

Bi-dimensional US and CFD examinations were performed using an ultrasound unit (iU22 ultrasound system, Philips Healthcare, Monza, Italy) equipped with different micro-convex, convex and linear array probes of different frequencies (2–9 MHz). For the CEUS examination, a linear array probe (3–9 MHz) was used. Contrast-enhanced US was performed by intravenously injecting 0.05 mL/kg (0.02 mL/lb) of a second generation US contrast medium (SonoVue, Bracco^®^ diagnostic, Milano, Italy) through an indwelling cephalic venous catheter, followed by a rapid immediate bolus of 5 mL saline. The arrival of the contrast medium at the level of the target organ was then visualized using dedicated imaging software of the ultrasound machine that selectively displaces the signal deriving only from the contrast medium, allowing perfect definition of the perfusion pattern of the region of interest. Bi-dimensional US, CFD and CEUS findings are summarized in Table 2.

**Figure 1:**
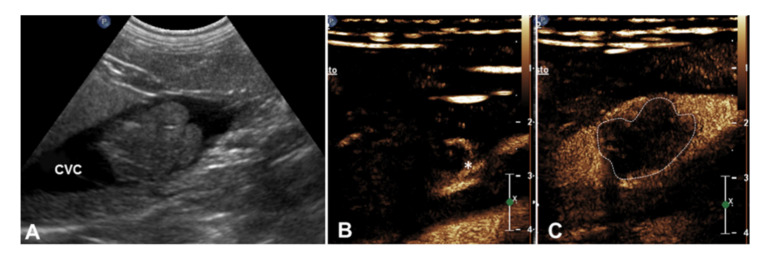
(**A**) Two-dimensional and (**B**,**C**) contrast-enhanced images of malignant thrombosis involving the caudal vena cava in a dog with right adrenal carcinoma (case 4). (**A**) Hyperechoic, solid thrombus is seen in the caudal vena cava (CVC), with a focal dilation of the vessel; (**B**) evidence of the enhancement of a tortuous newly formed vessel inside the thrombus (*), while caudal vena cava is still anechoic (Anticipated wash-in); (**C**) inhomogeneous wash out of the thrombus (dotted) while the contrast medium is still present on the lumen of the caudal vena cava (CVC).

Contrast-enhanced US was feasible in all dogs. In every case, contrast uptake from the thrombus was identified. Moreover, a feeding vessel was clearly visible inside the thrombus in two dogs (Figure 1).

**Figure 2:**
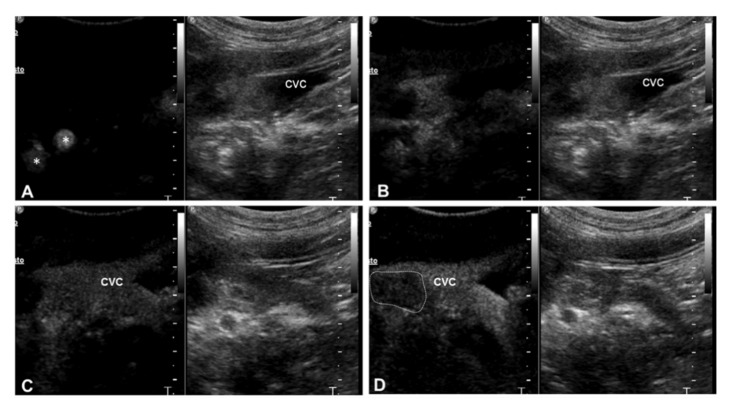
(**A**–**D**) Representative contrast-enhanced ultrasound sequences of malignant thrombosis involving the caudal vena cava in a dog with right adrenal carcinoma (case 3). Each sequence illustrates contrast enhancement on the left and the gray scale image on the right. (**A**) Left image shows the contrast medium in the celiac and mesenteric arteries (*) (Arterial phase). The grayscale image shows the thrombus in the vessel; (**B**) In the left image, a diffuse enhancement within the thrombus is seen, while caudal vena cava is still anechoic (Anticipated wash-in); (**C**) The contrast medium arrives in the lumen of the caudal vena cava and the thrombus is not detectable; (**D**) On the left image, the thrombus shows wash out (dotted lines) while the contrast medium is still present on the lumen of the caudal vena cava (CVC).

**Figure 3:**
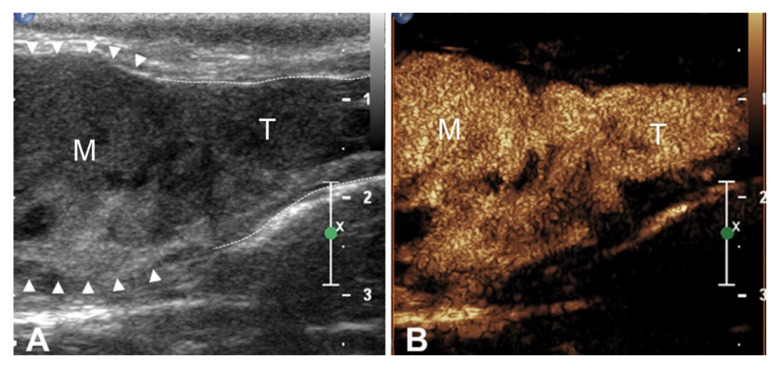
(**A**) Two-dimensional and (**B**) contrast-enhanced images of malignant thrombosis involving the thyroid in a dog with thyroid carcinoma (case 1). (**A**) The thyroid mass (arrows head) invades the lumen of the right thyroid vein (dotted); (**B**) contrast-enhanced ultrasound shows synchronous contrast uptake of the primary thyroid mass and the thrombus inside the thyroid vein.

The aspect of the uptake was homogeneous in the two cases originating from the thyroid and heterogeneous with large non-enhancing areas in all abdominal cases. The margination of the thrombus was regular and well-defined in two cases and irregular and ill-defined in the other five dogs. All thrombi showed an earlier contrast uptake compared to the timing of contrast medium arrival within the venous vessel (Figure 2). In 3 dogs, direct comparison of CEUS behavior of the primary tumor and the thrombus was possible on the same field of view. In every case, a simultaneous contrast uptake from the lesions was observed (Figure 3).

Two dogs (one thyroid mass, case 1; one adrenal mass, case 3) had surgical removal of the primary tumor and the thrombus. Histopathological diagnosis was consistent with solid-follicular thyroid carcinoma with neoplastic vascular invasion in the first case and with adrenocortical carcinoma associated with intravascular neoplastic thrombosis in the other one. Two dogs died few months after the US; necropsy showed a thyroid carcinoma with neoplastic invasion of the thyroid vein in one case (case 2) and a cortical adrenal gland carcinoma with neoplastic thrombotic caval invasion in the other (case 4). Necrotic areas within the tumor thrombus were detected at histology in one case (case 3) and endothelial invasion in another case (case 1).

In 3 cases the neoplastic mass extending into the caval vein was associated with multiple hepatic lesions detected on US. In these cases, cytology of the liver nodules suggested adrenal medullary neuroendocrine tumor (cases 5 and 6) and retroperitoneal liposarcoma (case 7) as primary diagnoses. Surgery was recommended, but declined by the owners; accordingly, histopathology of the intravascular mass was not available for these last three cases, and the diagnosis of tumor thrombi in these cases was only presumptive.

## 4. Discussion

In this study, CEUS could identify all tumor thrombi, whereas B-mode US allowed their visualization but could not establish the continuity with the primary tumor in every case. In addition, CEUS could better describe the vascularity of the tumor thrombi compared to CFD.

Conventional B-mode US is a fast and noninvasive tool to examine intra-abdominal structures, superficial neck structures, thyroid lobes and adjacent vessels. A recent study showed that conventional US is 100% sensitive and 96% specific in detecting vascular invasion of the caudal vena cava in dogs with adrenal gland tumors [11]. However, small thrombi or small vessels are difficult to accurately evaluate with US [11]. Moreover, neoplastic thrombosis and simple vascular thrombosis due to clot formation are difficult to differentiate on US [13].

Conventional B-mode US detected venous thrombosis in all patients of our study. Bi-dimensional features of tumor thrombi have been previously reported in literature and they are characterized by continuity between the primary mass and the thrombus [18,19] and dilation of the vessel in the site of thrombosis, which can be associated with rupture or infiltration of the vessel wall [20]. Only two of the seven thrombi were clearly connected with the primary mass, and dilation of the vessel was seen in four of the seven cases. This could be related to the position of the primary tumor itself, i.e., thyroid and adrenal glands that are smaller to image and require good knowledge of the regional anatomy (thyroid vessels), the mass effect on adjacent vascular structures or technically more challenging to reach because of the depth of the organ (adrenal) [21]. None of them presented visible rupture of the vessel wall.

Color-Doppler examination of the thrombus may be helpful in detecting intralesional signal due to presence of newly formed tumoral vessels, suggesting a neoplastic origin of the thrombus. However, this technique is limited by the low sensitivity of Doppler in detecting small vessels, low velocity blood flow and microvascularization in general [14]. In this case series, CFD was poorly sensitive in detecting thrombus vascularization in only two dogs was a Doppler signal evident into the thrombus. In humans with hepatocellular carcinoma, the detection of arterial flow within the thrombus using CFD is highly specific for a neoplastic nature of the thrombosis, but its sensitivity is low (20–51%) [13,14].

In human medicine, CEUS showed higher sensitivity in detecting vascularized tumor thrombosis when compared to CFD examination [14,15]. In humans, CEUS is also used to differentiate malignant thrombosis from a simple benign thrombus. In patients with hepatocellular carcinoma, CEUS shows high sensitivity (88–97.7%), specificity (100%) and accuracy (92.5–95.5%) in detecting and characterizing portal vein thrombosis, being the most sensitive imaging technique even when compared to contrast-enhanced computed tomography (CT) [13,14,17]. The intrinsic sensitivity of CEUS is higher than CT, as it allows single bubbles having the dimension of a red blood cell to be imaged and the contrast between contrast-enhanced blood and tissue is very high [22]. In addition, CEUS is performed in real time and allow to monitor continuously the contrast distribution, whereas in CT the selection of the vascular phase, i.e., the timing of the acquisition compared to the intravenous injection of the contrast agent, can influence the detection of thrombosis [15]. In small animals CT, the limited spatial resolution and the possible presence of streamlining artifact could reduce the possibility to correctly diagnose thrombosis [23], and CT may also be unable to detect endothelial invasion or evaluate the risk of fragmentations, both important features for a correct surgical planning [10].

In dogs, some studies have been carried out in order to determine the utility of contrast-enhanced CT in the detection of vascular luminal invasion by adrenal gland masses [12,24]. They showed high sensitivity and specificity of CT (respectively 92% and 100%) for detection of tumor thrombi, with an accuracy of 95%, and excellent agreement has been observed between CT signs of malignant thrombosis and vascular invasion found at surgery or necropsy [24]. Nevertheless, there is only one preliminary study in veterinary medicine comparing the use of US and/or CT for the evaluation of adrenal tumors in dogs and the detection of tissue invasion or thrombus [25]. Two studies described CEUS pattern of adrenal tumors in dogs [26,27], but a detailed description of the thrombus enhancement was not provided.

In our case series, contrast uptake of the thrombus was considered the main feature of malignancy, and it was present in all cases. Contrast-enhanced US may also clearly detect new-formed vessels within the thrombus. Studies in humans, however, reported that CEUS may misdiagnose a benign thrombus when some of its portion are revascularized, mimicking a vascularized malignant neoplastic thrombosis [14].

For this reason, other features were evaluated. Particularly, the timing of thrombus enhancement was always compared to the invaded vessel and in three cases to the primary neoplastic mass. Malignant thrombi showed a contrast uptake synchronous with the primary mass while the enhancement was always anticipated in the thrombus when compared to the invaded veins. This behavior is meant to be a consequence of newly formed arterial vessels inside the malignant thrombi (early arterial enhancement) that typically receive the contrast before it reaches the systemic venous system [13].

The aspect of the contrast uptake of the tumor thrombi was homogeneous in two dogs with thyroid tumors, while was heterogeneous, with several non-enhancing areas within the thrombus in the caval tumor thrombi. This aspect could reflect the presence of necrotic areas within the thrombus (histologically confirmed in one case). The necrotic areas within a tumor thrombus could lead to its fragmentation and predispose to pulmonary thromboembolism. For this reason, the possibility of a thrombus to fragment should be taken in account when deciding the treatment, in case of surgery in deciding the timing for the surgery and to formulate a prognosis.

Similarly, the possibility of CEUS to detect endothelial invasion could be extremely useful for surgical planning, as, for instance, a cavectomy is required in case of endothelial invasion from the neoplastic adrenal tissue. The irregular and ill-defined margination of the thrombus, with particular attention to the portions adjacent to the vessel walls, could reflect the endothelial invasion, as confirmed histologically in one of our cases.

This study has some limitations mainly related to its retrospective nature. First of all, the limited number of cases associated with the absence of dogs affected by benign thrombi prevented the possibility to evaluate the accuracy of CEUS in distinguishing benign from malignant thrombi. The lack of histological confirmation of thrombus malignant origin in three cases and confirmation of necrotic areas within the thrombus or endothelial invasion in only two cases are others limitations of this study. Further studies, including cases of nonneoplastic thrombi, are needed in order to confirm the peculiarity of CEUS features of malignant thrombi.

## 5. Conclusions

In conclusion, this is the first report describing the use of CEUS to characterize intravenous thrombosis in dogs with malignancies. On the basis of these preliminary experience, CEUS appeared to be easy to perform and useful for the detection of malignant intravascular invasion. In particular, the presence of an arterial-phase enhancement of the thrombus and a washout in the venous phase were consistently identified for the malignant thrombi characterized in this study.

## Figures and Tables

**Table 1:** Signalment and clinical signs of a population of dogs with neoplastic venous thrombosis.

Case No	Breed	Sex	Age (Years)	Weight (kg)	Clinical Symptoms	CBC and Biochemistry Abnormalities	Type of Tumor
1	Mixed	FS	10	27	Swelling of the neck, sudden change of voice	Elevated serum alkaline phosphatase	Thyroid carcinoma
2	Mixed	MN	11	14	Swelling of the neck, sudden change of voice	None	Thyroid carcinoma
3	Welsh Terrier	MN	7	15	PU/PD, polyphagia abdominal distension	Elevated serum alkaline phosphatase	Adrenal carcinoma
4	Mixed	F	10	9	Abdominal distension, diarrhea	None	Adrenal carcinoma
5	Boxer	M	11	32	PU/PD, diarrhea	Elevated serum alkaline phosphatase	Adrenal pheochromocytoma
6	Labrador	FS	13	32	PU/PD, polyphagia abdominal distension	Elevated serum alkaline phosphatase	Adrenal pheochromocytoma
7	Mixed	MN	13	33	PU/PD, diarrhea	Elevated serum alkaline phosphatase	Retroperitoneal liposarcoma

FS—female spayed; MN—male neutered; F—female intact; M—male intact; PU/PD—polyuria and polydipsia; CBC—complete blood count.

**Table 2:** Two-dimensional ultrasound (US), color flow Doppler (CFD) and contrast-enhanced ultrasonographic (CEUS) findings in a group of dogs with neoplastic venous thrombosis.

Case No	Site of the Primary Tumor	Thrombus Location	B-Mode US: Primary Tumor	B-Mode US: Thrombus	CFD: Thrombus	CEUS Findings of the Thrombus				
						Contrast Uptake	Aspect of the Contrast Uptake	Margination of the Thrombus	Enhancement Compared to the Vessel	Enhancement Compared to the Primary Tumor
1	Thyroid carcinoma	Thyroid veins (right and left)	2 Hyperechoic nodules (left: 2.8 cm; right 3 cm)	Intravascular homogeneous mass with vessel distension and contiguity with the primary tumor	Intralesional Doppler signal; residual flow within the lumen of the vessel	Present	Homogeneous	Irregular and ill-defined	Earlier	Simultaneous
2	Thyroid carcinoma	Left thyroid vein	1 Heterogeneous partially anechoic nodule on the left thyroid lobe (3 cm)	Intravascular homogeneous mass	Residual flow within the lumen of the vessel	Present	Homogeneous	Regular and well-defined	Earlier	Simultaneous
3	Right adrenal carcinoma	Caudal vena cava	1 Heterogeneous partially hyperechoic nodule with acoustic shadowing on the right adrenal gland (2.9 cm)	Intravascular homogeneous mass in contiguity with the primary tumor	Residual flow within the lumen of the vessel	Present	Heterogeneous	Irregular and ill-defined	Earlier	N/A
4	Right adrenal carcinoma	Caudal vena cava	Heterogeneous partially hyperechoic right adrenal gland with acoustic shadowing (width 1.5 cm)	Intravascular homogeneous mass with vessel distension	Residual flow within the lumen of the vessel	Present	Heterogeneous	Irregular and ill-defined	Earlier	N/A
5	Right adrenal pheochromocytoma	Caudal vena cava	1 Heterogeneous mass on the right adrenal gland (5 cm)	Intravascular homogeneous mass with vessel distension	Residual flow within the lumen of the vessel	Present	Heterogeneous	Regular and well-defined	Earlier	Simultaneous
6	Left adrenal pheochromocytoma	Caudal vena cava	Heterogeneous left adrenal gland (width 2.3 cm)	Intravascular homogeneous mass with vessel distension	Residual flow within the lumen of the vessel	Present	Heterogeneous	Irregular and ill-defined	Earlier	N/A
7	Retroperitoneal liposarcoma	Caudal vena cava	Heterogeneous mass on the left retroperitoneal space, involving the left adrenal gland (3.5 cm)	Intravascular homogeneous mass	Intralesional Doppler signal; residual flow within the lumen of the vessel	Present	Heterogeneous	Irregular and ill-defined	Earlier	NA
